# Intra-Ocular Pressure Response to Dexamethasone Implant Injections in Patients with a History of Filtering Surgery: The TRABEX Study

**DOI:** 10.3390/pharmaceutics14091756

**Published:** 2022-08-23

**Authors:** Amina Rezkallah, Laurent Kodjikian, Aymeric Barbarroux, Corentin Laventure, Antoine Motreff, Samuel Chacun, Frédéric Matonti, Philippe Denis, Thibaud Mathis

**Affiliations:** 1 Service d’Ophtalmologie, Hôpital de la Croix-Rousse, Hospices Civils de Lyon, 69004 Lyon, France; 2 UMR-CNRS 5510 Matéis, Université Lyon 1, 69100 Villeurbanne, France; 3 Centre Monticelli Paradis, 13008 Marseille, France; 4 CNRS, INT, Institut Neuroscience Timone, Univeristé Aix Marseille, 13005 Marseille, France; 5 Groupe Almaviva Santé, Clinique Juge, 13008 Marseille, France

**Keywords:** corticosteroids intravitreal injection, dexamethasone, chronic macular edema, intraocular pressure, glaucoma

## Abstract

Patients with both macular edemas, of various etiologies such as diabetes and glaucoma, may suffer serious loss of vision if either disease goes untreated. Where no effective alternative therapies are available, dexamethasone implant (DEX-I) injections may be the only choice of treatment, despite the risk of a possible increase in intraocular pressure (IOP) when using steroids. Although many studies have reported on IOP evolution in eyes treated with DEX-I, little is known specifically about eyes with a history of filtering surgery. The aim of this observational series was to evaluate the IOP response following DEX-I injection in eyes presenting conventional filtering surgeries or microinvasive glaucoma surgeries (MIGS). Twenty-five eyes were included in this study. A total of 64% of the eyes did not experience OHT during follow-up. Additional IOP-lowering therapy was needed for 32% of eyes, and 20% of eyes (all showing bleb fibrosis) required further filtering surgery: 50% of eyes in the MIGS group and 10.5% of eyes in the conventional filtering surgery group. A significant positive correlation was found between IOP at baseline and the maximum IOP throughout follow-ups after DEX-I (r = 0.45, *p* = 0.02). In conclusion, if DEX-I is used when there are no alternative therapies for treating macular edema, IOP in eyes with a history of filtering surgery is generally manageable. Those eyes which previously underwent conventional therapy with effective blebs obtained better IOP control after DEX-I injections and mostly did not require any additional IOP-lowering therapy or surgery.

## 1. Introduction

Dexamethasone implants (DEX-I) consist of a biodegradable copolymer of polyactic-co-glycolic acid containing micronized dexamethasone, which is injected in the intravitreal cavity for the treatment of macular edema of various etiologies, and especially diabetes [1]. However, the main ocular adverse effect of DEX-I is the rise of intra-ocular pressure (IOP) leading to ocular hypertension (OHT) and ultimately glaucoma [2]. Glaucoma is a progressive optic neuropathy resulting in visual field loss through retinal ganglion cell and axon degeneration in excess of physiological age-related changes and in the absence of any ocular or neurodegenerative pathologies [3]. OHT being the main risk factor for glaucoma, the strict control of IOP below a determined threshold is thus a goal to be reached in this respect [4]. Although IOP-lowering therapies are effective as a means to reduce the risk of glaucoma progression, some patients require laser or invasive filtering surgery to reduce IOP [5,6]. In recent years, microinvasive glaucoma surgeries (MIGS) have been proposed but still need to be studied on long-term follow-up [7].

OHT resulting from DEX-I intravitreal injections is usually successfully managed with IOP-lowering therapy [3,4]. However, some cases of high-responders have been reported with DEX-I, with an increase in IOP of more than 15 mmHg according to Becker’s classification [2,8,9]. Steroid-induced OHT results from the inhibition of proteases and phagocytosis of the trabecular cells, which reduces damage to the extracellular matrix of the trabecular meshwork [10,11]. These modifications to the microstructure of the trabeculum induce an increased resistance to aqueous humor drainage, leading to reduced filtration and consequent OHT [10,11]. Therefore, it is not recommended to reinject high-responder patients, or patients with glaucoma under dual or more IOP-lowering therapies [12,13]. However, glaucomatous patients can also suffer from a chronic macula edema that can lead to permanent vision loss if untreated. In cases where no effective alternative therapies are available or efficient, steroids may remain the only option for managing the edema [14,15]. Our team has already reported that patients who underwent the XEN^®^ gel stent procedure for DEX-I high-responders had well-controlled IOP in the short term after DEX-I reinjection [16]. However, no data are currently available in the literature as regards DEX-I injections following conventional filtering surgeries or long-term IOP following MIGS.

The aim of present study, which evaluates the IOP response to DEX-I injections after conventional filtering surgeries and MIGS procedures, was to collect data to fill this gap.

## 2. Materials and Methods

### 2.1. Study Design and Population

An observational, retrospective, monocentric, consecutive series was conducted in the Ophthalmology department of the Croix-Rousse University Hospital in Lyon, France. A database search was performed to include eyes with filtering surgeries which then received DEX-I intravitreal therapy between January 2017 and December 2021. All the filtering surgeries were associated with subconjunctival injection of 0.2 mg/mL mitomycin. This research was conducted in accordance with the Declaration of Helsinki. The informed consent of the patients was obtained, and a local Ethics Committee (Hospices Civils de Lyon) approved the study registered under number 20-156.

### 2.2. Data Collection

**Figure 1:**
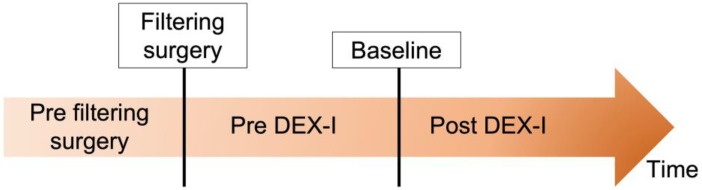
Study timeline. Baseline was considered as the time at which the first dexamethasone implant (DEX-I) following filtering surgery was injected.

All data were collected by reviewing patients’ medical charts. The following information was obtained for each patient: age, gender, medical history, number of DEX-I, number and type of filtering surgery, and surgical complications. Filtering surgeries comprised on the one hand of MIGS surgery, and on the other hand conventional surgery including trabeculectomy and non-penetrating deep sclerectomy (NPDS) (associated or not with secondary goniopuncture). Information on the patient’s IOP history was reported along with any eventual IOP-lowering therapy or surgery. Bleb conjunctival fibrosis was defined as subconjunctival fibrous tissue formation during postsurgical healing [17]. Baseline was considered as the time at which the first DEX-I was injected following filtering surgery (Figure 1).

### 2.3. Outcome Measures

The main outcome measure was the change in IOP following DEX-I injection. Secondary outcome measures were the incidence of OHT, the need for an additional lowering therapy, and the need for a subsequent glaucoma surgical procedure.

OHT was defined as IOP ≥ 25 mmHg and/or an increase of 10 mmHg over the follow-up period compared with baseline IOP [8]. Bleb fibrosis was defined by an increase of IOP without any internal ostium obstacle. Subgroup analyses included patients with filtering surgeries which included only MIGS procedure, in comparison to patients who underwent at least one conventional surgery.

### 2.4. Statistics

The description of the categorical variables was based on absolute frequencies (size) and relative frequencies (percentage). All quantitative variables are described using the mean and standard deviation (SD). A paired Wilcoxon signed-rank test was used to evaluate the differences in IOP measured at the baseline and at the IOP peak during the follow-up. Spearman correlation was used to evaluate the correlation between baseline IOP and maximum IOP following DEX-I injections. A *p*-value < 0.05 was statistically significant. Graph prism version 9.2 (GraphPad Software, San Diego, CA, USA) was used for the data analysis and graphic presentation.

## 3. Results

### 3.1. Glaucoma/OHT History

A total of 25 eyes from 21 patients were included. At baseline, the mean age was 68.5 years (13.6) and 42.9% of patients were women. The main indications for filtering surgery were primary open-angle glaucoma (24%), chronic angle-closure glaucoma (16%), and steroid-induced glaucoma (16%) (Table 1).

Before the first filtering surgery, mean (SD) IOP was 27.5 (10.8) mmHg. The mean (SD) number of IOP-lowering therapies was 2.8 (1.2) molecules. In detail, 12% of eyes were treated with one molecule, 20% with dual IOP-lowering therapy, 20% with triple IOP-lowering therapy, and 48% with quadruple IOP-lowering therapy. In addition, 52% of eyes were also treated with oral acetazolamide. Two eyes had benefited from previous selective laser trabeculoplasty.

The mean (SD) number of filtering surgeries before baseline was 1.2 (0.6) (min–max, 1–3). Three eyes underwent more than one filtering surgery before baseline.

The last filtering surgery before baseline was MIGS (XEN^®^ gel stent for all) in 6 eyes (24.0%) or conventional filtering surgery in 19 eyes (76.0%), including trabeculectomy for 12 eyes (48.0%), and NPDS for 7 eyes (28.0%). 85.7% of eyes who had NPDS underwent goniopuncture during follow-up. An XEN^®^ gel stent was the only MIGS used before baseline.

### 3.2. Patient Population and Disease Characteristics at Baseline

The mean (SD) time between filtering surgery and DEX-I injection was 4.6 (3.8) months. Eight eyes (31%) had a macular edema before the filtering surgery and underwent a DEX-I injection during the filtering surgery. Mean (SD) IOP at baseline was 11.0 (3.7) mmHg. Nine eyes (36%) were treated with IOP-lowering therapy. Mean (SD) IOP-lowering therapy was 0.3 (0.7): 16 eyes (64%) had no treatment, 3 eyes (12%) were treated with singular IOP-lowering therapy, 2 eyes (8%) with dual IOP-lowering therapy, 3 eyes (12%) with trial IOP-lowering therapies and 1 eye (4%) with quadral IOP-lowering therapies. One eye (4%) was treated with oral acetazolamide (Table 2).

### 3.3. IOP Profile following DEX-I Injections

**Figure 2:**
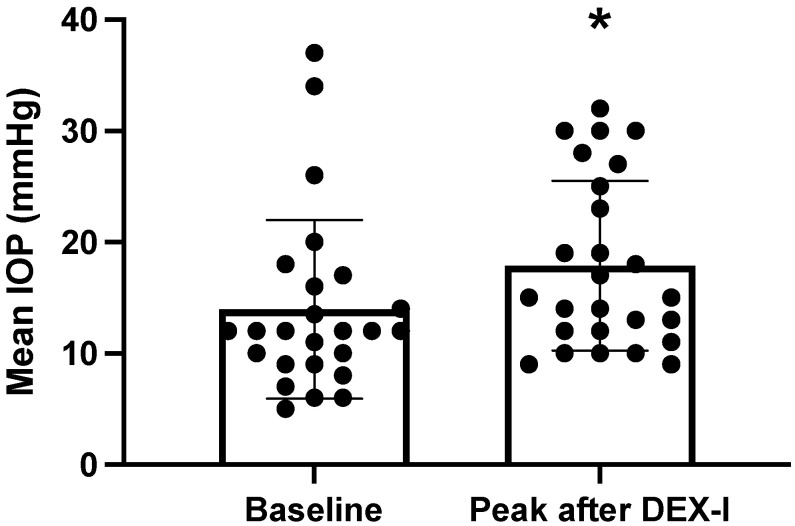
Comparison of intraocular pressure (IOP) at baseline and at the time of IOP peak (Wilcoxon signed-rank test * *p* = 0.02).

**Figure 3:**
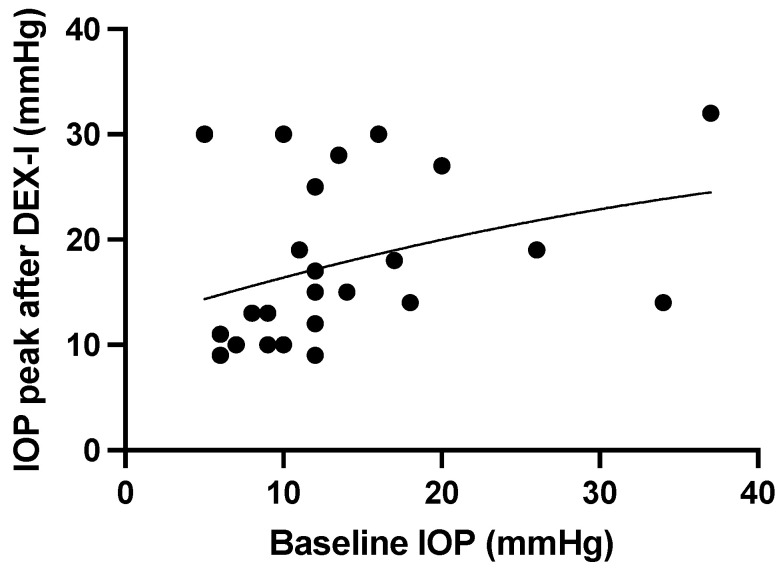
Maximal intraocular pressure (IOP) after DEX-I injection according to baseline IOP (r = 0.45, *p* = 0.02).

The main indications for DEX-I treatment were diabetic macular edema (DME) in 10 eyes (40%), uveitis in 7 eyes (28%), retinal vein occlusion in 4 eyes (16%), and post-surgery macular edema in 4 eyes (16%). The mean (SD) number of DEX-I injections following filtering surgery was 3.2 (3.3) (min–max: 0–13) during a mean (SD) follow-up period of 27.2 (15.1) months. IOP peak was detected in a mean (SD) time of 2.9 (1.2) months during follow-up (min–max: 0.2–4). There was a significant increase in mean (SD) IOP between baseline and maximum IOP (11.0 (3.7) mmHg versus 19.2 (7.8) mmHg, respectively, *p* = 0.03, Figure 2). There was a significant correlation between baseline IOP and maximum IOP following DEX-I injections (r = 0.45, *p* = 0.02, Figure 3). The mean (SD) number of DEX-I at the time of IOP peak was 1.5 (1.5) (min–max: 1–7).

A total of 9 eyes (36%) had OHT after a mean (SD) number of DEX-I of 1.4 (1.0) (min–max: 1–4), and an additional lowering-therapy was needed to control IOP in 8 eyes (32%). Among these, 2 eyes (25%) needed a dual IOP-lowering therapy, and 6 eyes (75%) needed a trial IOP-lowering therapy. Oral acetazolamide was introduced to control IOP in 4 eyes (50%). For 6/8 eyes (75%), the IOP-lowering therapy was modified after the first DEX-I. For 2/8 eyes (25%) the reintroduction of the IOP-lowering therapy was delayed until after 2 and 6 DEX-I, respectively. A revision of the surgery was performed on 8 eyes (32%). Five eyes (20%) required at least one additional filtering surgery: 3/6 eyes (50%) treated with MIGS (XEN^®^ gel stent), and 2/19 eyes (10.5%) which received the additional filtering surgery procedure (Table 3). All these eyes showed bleb fibrosis before DEX-I injection. The mean (SD) number of DEX-I before the second filtering surgery was 3.8 (2.2). A total of 3 eyes (60%) of those requiring additional surgery had at least one DEX-I after the second filtering surgery. Two eyes required ≥2 additional surgeries.

### 3.4. Filtering Surgery for Steroid High-Responder Eyes

A total of 13 eyes (52%) were treated with DEX-I before filtering surgery. The mean (SD) age in this subgroup was 69.9 years (9.8). The overall mean (SD) number of DEX-I before the surgery was 2.8 (2.5) (min–max: 1–9). The filtering surgeries were MIGS in 5 eyes (38.5%) and conventional therapy in 8 eyes (61.5%), including trabeculectomy for 3 eyes (23%), and NPDS for 5 eyes (38.5%). The mean (SD) time between filtering surgery and DEX-I reinjection was 5.4 (2.7) months.

The mean (SD) number of DEX-I after baseline was 4.6 (3.8) (min–max: 1–13), during a mean (SD) follow-up of 32.6 (17.5) months. The mean peak in (SD) IOP after baseline was 18.5 (7.9) mmHg and was detected in a mean (SD) time of 2.3 (1.2) months. The mean (SD) number of DEX-I at the time of IOP peak was 3.0 (2.5) (min–max: 1–6).

A total of 6 eyes (46.2%) had OHT (3/5 XEN^®^ gel stent, 0/3 trabeculectomy, 3/5 NPDS) after a mean (SD) number of DEX-I of 1.8 (1.5) (min–max: 1–4). The 5 eyes in the NPDS group underwent goniopuncture and 4 of these eyes (80%) did not require any additional IOP-lowering therapy.

Additional IOP-lowering therapy was needed for 4 eyes (30.8%) (3/5 XEN^®^ gel stent and 1/5 NPDS). All these eyes required triple IOP-lowering therapy. Oral acetazolamide was not introduced for any eyes. For 3 eyes (75.0%), the IOP-lowering therapy was introduced/modified after the first DEX-I injection and for one eye the reintroduction of an IOP-lowering therapy was delayed. A revision of the surgery was performed for 3 eyes (23.8%) (all in the MIGS group), and 3 eyes (23.8%) required additional filtering surgery (2 eyes in the MIGS group, and 1 eye in the NPDS group).

## 4. Discussion

This study aims to analyze IOP response after DEX-I injections in patients with a history of filtering surgery. Although it has been already shown that OHT occurs after DEX-I for patients without glaucoma and more frequently in glaucomatous population, the increase in IOP appears to be easily managed with IOP-lowering therapy in 97% of eyes [2,8]. The SAFODEX study, which is the largest cohort study on DEX-I safety, reported that patients with glaucoma or a history of OHT, initially on dual-or triple-combination therapies, had poor pressure tolerance after DEX-I injections [8]. However, no data were reported on patients who had already undergone filtering surgery before DEX-I injection. In fact, only a small part (about 1 mm) of the trabeculum is removed or opened during filtering surgery, and the remaining trabeculum can be pathologic and lead to a decreased filtration flow. Moreover, there are cases of bleb fibrosis or obstacles in the trabeculum ostium that can also reduce the long-term success of the surgery. As a result, an eye could develop ocular hypertension after DEX-I injection even after a conventional filtering surgery.

In the present study, we showed that DEX-I injections are safe and can be continued in patients with a history of filtering surgery. Approximately two-thirds of the eyes did not experience OHT during follow-up, 32% (8/25) required additional IOP-lowering therapy, in which 20% (5/25) underwent further filtering surgery after DEX-I: 50% of eyes in the MIGS group and 10.5% of eyes the in conventional filtering surgery group. However, in the latter case, DEX-I could be continued in some eyes due to better IOP control.

We found a significant positive correlation between initial IOP at baseline and maximum IOP over the duration of follow-up: eyes with higher baseline IOP experienced a higher increase in IOP after DEX-I. This correlation is further supported by the lower rate of OHT following DEX-I in the conventional surgery group, which is known to better control IOP than MIGS [18]. This difference is also illustrated by the higher rate of additional IOP-lowering therapy and the stronger need for further filtering surgery in eyes with XEN^®^ gel stents, despite good starting efficacy [16]. At the end of follow-up, half of the eyes treated with MIGS required additional filtering surgery in comparison with approximately 10% of eyes treated with conventional filtering therapy. Most importantly, all eyes requiring additional IOP-lowering surgery showed bleb fibrosis before DEX-I injections, highlighting the need for careful examination before indicating the intravitreal treatment. Moreover, it should be noted that most patients (85.7%) who had NPDS in the present cohort also underwent goniopuncture during follow-up, as it has been shown to decrease IOP significantly for at least 5 years [19].

We also found that the IOP peak occurred at a mean of 2.9 months for all the groups, explaining that eyes requiring additional IOP-lowering therapy generally did so after the first DEX-I injection. The predictable nature of the IOP response after the first DEX-I has been previously demonstrated. In the SAFODEX study, most eyes with OHT had an IOP peak after the first or second DEX-I injection, regardless of whether they were glaucomatous or not [8]. Another study by Zarranz-Ventura et al. confirmed that repeat injections of DEX-I do not increase the risk of IOP elevation [20].

Very few studies have reported outcomes for patients injected by DEX-I after filtering surgery. Vié et al. found that the 5 patients included in their study with a history of conventional filtering surgery had a profile close to that of the control patients (33% of patients required an additional IOP-lowering therapy) [21]. Another study reported that IOP in 2 patients who required additional treatment with DEX-I after combined phakosclerectomy surgery was kept under control without treatment [22]. Moreover, our team has previously reported the short-term efficacy of XEN^®^ gel stent on IOP when treating DEX-I induced OHT [16].

The subgroup of patients who underwent filtering surgery due to a high response to steroids continued to be treated with DEX-I after the IOP-lowering procedure due to the lack of therapeutic alternatives. Although some eyes (30.8%) required an additional IOP-lowering procedure, these were generally not those with a history of conventional therapy. These results raise the possibility of reinjecting some eyes with DEX-I despite a history of steroid-induced OHT. This is particularly interesting in eyes which do not respond to anti-VEGF treatment and where only DEX-I is effective, as is the case for 30% of DME patients and 20–30% of RVO patients [14,23,24]. Furthermore, in the context of OHT or established glaucoma, anti-VEGF drugs are not completely risk-free [25]. Thus, in the event of ineffectiveness of these treatments in glaucoma patients, it is more logical not to neglect the use of corticoids.

The main limitation of the present study was its retrospective design, which limits the regularity of follow-up and the systematic reporting of data. Furthermore, the low number of patients included with this non-rare condition highlights the reluctance of retinal specialists to propose DEX-I treatment to patients with a history of filtering surgery. This explains heterogeneity in patient characteristics at baseline, as emphasized by the number of patients who underwent the filtering surgery at the same time as DEX-I injection for concomitant glaucoma and macular edema. Moreover, as a retrospective pilot study exploring an uncommon condition, we decided to include in analyses both eyes of patients with bilateral DEX-I injections to avoid a potential waste of information leading to less precise estimates of effect and less power [26]. This statistical methodology could induce a bias as it can violate the assumption of the independence of observations. However, it has been previously showed that both eyes generally respond differently to DEX-I in terms of OHT [2]. This is the only study that provides a specific analysis of the IOP response to DEX-I in this population of patients, and further larger scale studies are needed to confirm our results. Another limitation is that the MIGS group only covers the XEN^®^ gel stent procedure, and not new therapeutic options such as iStent^®^ or Preserflo^®^. Finally, although follow-up was longer than one year, our results should be confirmed by longer studies.

In conclusion, we show here that in cases where there are no alternative therapies for treating macular edema in patients with a history of filtering surgery, DEX-I could be proposed with generally manageable IOP. Patients who previously underwent conventional therapy with effective blebs obtained better IOP control after DEX-I injections and mostly did not require additional IOP-lowering therapy or surgery.

## Figures and Tables

**Table 1:** Patient characteristics at baseline.

Patient Characteristics	25 Eyes (21 Patients)
Mean age, years (SD)	68.5 (13.6)
Sex Female, *n* (%)	9 (42.9)
Previous ocular surgery, *n* (%)	
Pseudophakic	22 (88)
Vitrectomy	3 (12)
Indication of filtering surgery, *n* (%)	
POAG	6 (24)
Chronic angle-closure glaucoma	4 (16)
Steroid-induced glaucoma	4 (16)
Steroid-induced OHT without glaucoma	6 (24)
Secondary uveitic glaucoma	4 (16)
Pseudoexfoliative glaucoma	1 (4)

SD: standard deviation; OHT: ocular hypertension; POAG: primary open-angle glaucoma.

**Table 2:** Filtering surgery and IOP-disease characteristics at baseline.

Characteristics at Baseline	MIGS	Conventional Filtering Surgery		
	XEN^®^ Gel Stent	Total	Trabeculectomy	NPDS
Eyes, *n* (%) Complications, *n* (%)	6 (24%) Fibrosis: 3 (50) Exteriorization: 1 (16.7%)	19 (76) Fibrosis: 4 (21.1%)	12 (48) Fibrosis: 2 (16.7%)	7 (28) Fibrosis: 2 (28.6%)
Mean IOP, mmHg (SD) IOP-lowering therapy, *n* (%) Mean time since filtering surgery, months (SD)	9.4 (2.4) 2 (33.3%) 1.2 (0.4)	14.7 (8.9) 5 (26.3%) 3.45 (3.9)	11.3 (6.1) 3 (25%) 4.9 (4.2)	21.8 (11.0) 2 (28.6%) 0.3 (0.8)

SD: standard deviation; IOP: intraocular pressure; MIGS: microinvasive glaucoma surgery; NPDS: non-penetrating deep sclerectomy.

**Table 3:** IOP profile post dexamethasone implant injections according to types of filtering surgery.

Characteristics at Baseline	MIGS	Conventional Filtering Surgery		
	XEN^®^ Gel Stent	Total	Trabeculectomy	NPDS
Eyes, *n* (%)	6 (24%)	19 (76)	12 (48)	7 (28)
Incidence of OHT, *n* (%)	4 (66.7)	4 (21.1)	3 (25.0)	1 (14.3)
Additional IOP-lowering therapy, *n* (%) Revision of filtering surgery, *n* (%) Additional filtering surgery, *n* (%)	4 (66.7) 3 (50.0) 3 (50.0)	4 (21.1) 5 (26.3) 2 (10.5)	3 (25.0) 3 (25.0) 1 (8.3)	1 (14.3) 2 (28.6) 1 (14.3)
Mean follow-up from baseline, months (SD)	34.5 (15.7)	15.1 (3.9)	17.8 (11.4)	8.0 (5.2)

SD: standard deviation; IOP: intraocular pressure; MIGS: microinvasive glaucoma surgery; NPDS: non-penetrating deep sclerectomy.

## Data Availability

All data can be obtained upon request to the corresponding author.
